# Patient experience and satisfaction with symptomatic faecal immunochemical testing: an explanatory sequential mixed-methods evaluation

**DOI:** 10.3399/BJGP.2022.0241

**Published:** 2023-01

**Authors:** Natalie Gil, Helen Su, Kirandeep Kaur, Michael Barnett, Anna Murray, Stephen Duffy, Christian von Wagner, Robert S Kerrison

**Affiliations:** Department of Behavioural Science and Health;; Faculty of Medical Sciences, University College London, London.; Department of Behavioural Science and Health;; Cheshire and Merseyside Cancer Alliance, Cheshire and Merseyside.; Cheshire and Merseyside Cancer Alliance, Cheshire and Merseyside.; Centre for Prevention, Detection and Diagnosis, Wolfson Institute of Preventive Medicine, Queen Mary University London, London.; Department of Behavioural Science and Health;; School of Health Sciences, University of Surrey, Surrey.

**Keywords:** colorectal cancer, colorectal neoplasms, faecal immunochemical testing, multivariate analysis, patient experience, patient satisfaction, personal satisfaction, two-week-wait

## Abstract

**Background:**

Recent evidence suggests that faecal immunochemical testing (FIT) can rule out colorectal cancer (CRC) in symptomatic adults. To date, there has been little research exploring experiences of FIT for this population.

**Aim:**

To explore patient experience and satisfaction with FIT in an ‘early adopter’ site in England.

**Design:**

Explanatory sequential mixed-methods approach combining mailed quantitative surveys with semi-structured telephone interviews.

**Method:**

Multivariate logistic regression was used to analyse quantitative data. Thematic analysis was used to assess qualitative transcripts.

**Results:**

The survey had 260 responders, and it found that satisfaction with FIT was high (88.7%). Compared with test satisfaction, the proportion of responders satisfied with their GP consultation and how they received their results was lower (74.4% and 76.2%, respectively). Multivariate analysis showed that increased area-level deprivation and not receiving an explanation of the purpose of the test were associated with lower satisfaction with the GP consultation (both *P*-values <0.05), while increased area-level deprivation and not receiving results from the GP were associated with lower satisfaction with receiving results (both *P*-values <0.05). Interviews with responders (*n* = 20) helped explain the quantitative results. They revealed that ‘not knowing the purpose of the test’ caused ‘anxiety’ and ‘confusion’, which led to dissatisfaction. ‘Not receiving results from GP’ was considered ‘unacceptable’, as this left patients with a ‘niggling doubt’ and lack of diagnosis or assurance that they did not have cancer.

**Conclusion:**

Patient satisfaction with symptomatic FIT is high. Efforts to improve satisfaction should focus on ensuring that patients understand the purpose of the test and always receive their test results.

## INTRODUCTION

Despite improvements in colorectal cancer (CRC) survival during the last decade, the UK lags behind many other high-income countries, with just 64.2% of patients surviving for 5 or more years after diagnosis in the UK, compared with 73.3% in Australia.^1^ One possible explanation for the differences in 5-year survival between countries is the diagnostic interval (that is, the time from when a patient first presents to a GP to when they receive a diagnosis),^2^ which is significantly longer in the UK than in Denmark, Norway, and Australia.^3^ It has been acknowledged that primary care plays a major role across the cancer continuum,^3^ and such findings suggest a need for additional or improved diagnostic pathways to reduce primary care intervals and improve time to diagnosis and treatment.

The National Institute for Health and Care Excellence (NICE) diagnostic (DG30) and referral (NG12) guidelines for suspected CRC were revised in 2017 to include faecal immunochemical testing (FIT; an immunoassay-based test that measures the amount of haemoglobin [Hb] present in faeces) for individuals who have low-risk unexplained symptoms (but not rectal bleeding).^4^^,^^5^ The revisions represent a change for low-risk patients within the ‘two-week-wait’ (TWW) pathway for CRC, which has been in place since the year 2000, and requires patients with unexplained symptoms, suggestive of CRC, to be offered a colonoscopy within 2 weeks.^6^

The decision to include FIT in the pathway is supported by increasing evidence that it can be used as a rule-out investigation for suspected CRC (around 75% of symptomatic individuals can avoid colonoscopy when FIT is used to triage patients in the primary care setting.^7^^,^^8^ The test is widely regarded as cheap and convenient for the patient, as it can be completed in their own home^4^ and allows colonoscopy resources to be used more efficiently (most colonoscopies do not result in a CRC diagnosis in symptomatic individuals, and, owing to multiple factors including an increased burden of gastrointestinal disease and a decreased workforce capacity, there is rising demand for endoscopic procedures).^9^ This is particularly pertinent at present, as there is now significant constraint on NHS resources, and, following disruption brought about by the COVID-19 pandemic, increased wait times for colonoscopy.^10^ Moreover, the introduction of FIT has potential to allocate cancer service resources more efficiently, which may favourably impact the TWW referral system, allowing patients to be directed as appropriate based on their CRC risk.

As with the introduction of any new primary care pathway, successful implementation is key to ensuring maximum benefit to the patient.^11^ Furthermore, patient satisfaction with new tests in primary and secondary care is crucial to maintaining public trust and ensuring future visitations and help-seeking behaviour (which in turn can impact early diagnosis and cancer outcomes).^12^ As sites in the UK are beginning to implement symptomatic FIT pathways, patient satisfaction needs to be monitored and adjusted to provide guidance on further development of the pathway. The present study set out to evaluate patient experience and satisfaction with FIT as a triaging test for symptomatic patients at one of the UK’s ‘early adopter’ sites.

**Table:** How this fits in

In England, primary care now offers faecal immunochemical testing to symptomatic patients. This was introduced during the COVID-19 pandemic and was helpful in triaging patients when colonoscopy capacity was reduced. In this study, patient satisfaction with the pathway was evaluated at one of the ‘early adopter’ sites. The findings show that most patients are satisfied with the test itself but are less satisfied with the GP consultation and delivery of results (both being particularly evident in more deprived areas). The findings suggest that, given the test is related to cancer, patients should be informed of negative results as well as positive ones.

## METHOD

### Design

To explore patient experience and satisfaction with the symptomatic FIT pathway, an explanatory sequential mixed-methods approach was employed, comprising patient surveys (Study 1) and semi-structured interviews (Study 2).

### Setting

Both studies took place in Cheshire and Merseyside, which is disproportionately affected by CRC compared with the rest of England (the incidence rate for CRC has increased by 6% over the past decade within the region compared with 2% on average nationally).^13^

### Study 1: Patient surveys

All patients in Cheshire and Merseyside (*n* = 1571) who were offered a symptomatic FIT between 5 April and 19 July 2021 were sent a mailed survey (Supplementary Appendix S1) via Docmail (a third-party mailing company), 2–3 weeks after being sent a FIT kit (requested by their GP). For the purposes of the survey, the symptomatic FIT pathway was divided into three components, and patient satisfaction was assessed for each. A Likert scale was subsequently used to measure patient satisfaction with the GP consultation, completing the FIT kit, and receiving the FIT results. Descriptive statistics were used to report the demographic characteristics of the sample, as well as the number and percentage of participants satisfied with each aspect of the pathway (that is, ‘satisfaction with GP consultation’, ‘satisfaction with completing the test’, and ‘satisfaction with receiving results’). Area-level deprivation was determined using participant postcodes and the English Indices of Multiple Deprivation (IMD).^14^ IMD deciles were converted into tertiles, with deciles 1–3 indicating the least deprived, 4–6 mid-range, and 7–10 the most deprived. Experiential aspects, associated with different stages of the pathway (for example, whether the purpose of the test was explained) were assessed using either a single item or a series of items that made up a scale (for example, the clarity of the test kit instructions [the internal reliability of which was determined using Cronbach’s alpha; all scales had scores of 0.88 or greater]). Associations between patient satisfaction measures, demographic characteristics, and experiential aspects of the pathway were assessed using univariate and multivariate logistic regression (separate models were produced for ‘satisfaction with GP consultation’, ‘satisfaction with completing the test’, and ‘satisfaction with receiving results’).

Multiple imputation using five iterations was conducted to account for variables with >5% and <28.5% missing data (data completeness was >95% for 50% of variables; no variables had >28.5% missing data). The final models were derived by fitting a regression model, including all confounders, and combining estimates (from the five iterations) using Rubin’s rules.^15^ Sensitivity analysis was conducted by comparing the original dataset with the imputed data. There were no substantive differences between iterations (see Open Science Framework for original data and models, https://osf.io/tv23f/). Associations were considered statistically significant if the *P-*value was ≤0.05. All analyses were performed using SPSS statistics (version 27.0).

### Study 2: Patient interviews

To help explain associations between covariates and patient satisfaction, all individuals who received the survey were invited to participate in a one-to-one, in-depth, semi-structured telephone interview (see Supplementary Appendix S2 for interview schedule). Individuals who consented were subsequently contacted via their preferred method (email or telephone) shortly after receipt of their questionnaire. Individuals were excluded if they no longer wished to participate in a follow-up interview or if they withdrew consent during/after the interview. A semi-structured topic guide based on the survey responses was prepared ahead of the interviews.

All Interviews took place between July and August 2021 and were conducted via telephone by a female postgraduate student and a male postdoctoral researcher and lecturer. To ensure interesting free-text responses to the questionnaire were followed up during the interview, the interviewers had the participants’ survey responses to hand, and brought these into the interview as and when they were deemed relevant. In the first instance, 12 participants were interviewed. Transcripts from the interviews were then analysed using thematic analysis,^16^ and additional interviews were conducted and analysed in sets of four, with data gathering continuing until no new codes were identified following four consecutive interviews. This approach resulted in a total of 20 interviews being conducted, as no new codes were identified following the analysis of participants 17, 18, 19, and 20 (all transcripts were analysed independently by a postgraduate researcher; 30% were second-coded by another researcher). Conversations lasted up to 1 hour, were audio-recorded, transcribed verbatim by K International (a third-party transcription service), and then anonymised. Analytical themes were then developed from the codes through an iterative process of reflection on and interpretation of the coded text. Descriptive themes were shared with all authors to ensure they were consistent and apposite.

Qualitative and quantitative data were integrated using an advanced convergent meta-integration approach,^17^ in which quantitative and qualitative data were first synthesised and analysed separately. Then they were integrated and analysed together, using a ‘joint display’ of the qualitative and quantitative findings in an Excel spreadsheet, to identify any differences or similarities between the quantitative and qualitative findings, and determine whether the two sets of findings augmented one another (see Open Science Framework for worked example, https://osf.io/tv23f/).

The study protocol, survey, and interview schedule were reviewed and approved by the local audit and quality improvement team for Cheshire and Merseyside (that is, St Helens and Knowsley NHS Trust).

## RESULTS

### Study 1: Patient surveys

Of 1571 adults eligible to participate, 260 (16.5%) completed and returned a survey. The mean age of participants was 69.8 years (range 31–98), and the mean IMD tertile of the population was 2.13 (range 1–3). The majority of participants were female (*n* = 148, 56.9%), identified as white British (*n* = 243, 93.5%), and were born in the UK (*n* = 234, 90.0%). These figures are comparable to the wider populations of St Helens and Knowsley, which have low immigration and are less ethnically diverse than the rest of England.^18^ However, 46.9% (*n* = 122) of participants had obtained a GCSE or O Level (Grade A–C) or higher qualification, while 84.2% (*n* = 219) owned one or more cars and/or vans. These figures would indicate that the sample was more educated and of higher socioeconomic status than the local average, as St Helens is the eighth most deprived borough in England in terms of relative Health Deprivation and Disability,^19^ while Knowsley is the second most deprived borough in England^18^ (see Tables 1 and 2 for descriptive statistics and experiential items).

**Table 1.** Sample characteristics of survey participants (*n* = 260)

**Age (*n*=** **255)**	**Mean (Range)**
Age in years (continuous)	69.79 (31 98)
Missing	5

**Gender (*n*=** **256)**	***n* (%)**
Male	108 (41.54)
Female	148 (56.92)
Missing	4 (1.54)

**Ethnicity (*n* =** **255)**	***n* (%)**
White British	243 (93.46)
Any other ethnicity	12 (4.62)
Missing	5 (1.92)

**Education (*n* =** **216)**	***n* (%)**
O Level or GCSE (Grade D G) or lower qualification	94 (36.00)
O Level or GCSE (Grade A C) or higher qualification	122 (47.00)
Missing	44 (17.00)

**Area-level deprivation (*n*=** **231)**	***n* (%)**
IMD tertile 1 (continuous)	65 (25.00)
IMD tertile 2	71 (27.00)
IMD tertile 3	95 (37.00)
Missing	29 (11.00)

**Migrant status (*n* =** **250)**	***n* (%)**
Born in the UK	234 (90.00)
Emigrated to the UK	16 (6.00)
Missing	10 (4.00)

**Car ownership (*n* =** **256)**	***n* (%)**
No car or van	37 (14.00)
One or more car and or van	219 (84.00)
Missing	4 (2.00)

*IMD = Indices of Multiple Deprivation.*

**Table 2.** Descriptive statistics of psychological variables (*n* = 260)

**Did your GP explain the purpose of the test to you?**	***n* (%)**
No/Don’t know	39 (15.00)
Yes	217 (83.46)
Missing	4 (1.54)

**Did the GP provide instructions on how to complete the test?**	***n* (%)**
No/Don’t know	101 (38.85)
Yes	156 (60.00)
Missing	3 (1.15)

**Did the GP tell you how long the test would take to arrive?**	***n* (%)**
No/Don’t know	101 (38.85)
Yes	156 (60.00)
Missing	3 (1.15)

**Instruction scale**	**Mean (SD)**
-	14.72 (1.86)

**Collection scale**	**Mean (SD)**
-	17.05 (2.65)

**Doing the test made me feel anxious.**	***n* (%)**
Disagree/Strongly disagree	145 (55.77)
Agree/Strongly agree	95 (36.54)
Missing	20 (7.69)

**The thought of an abnormal result from the test scared me.**	***n* (%)**
Disagree/Strongly disagree	83 (31.92)
Agree/Strongly agree	163 (62.69)
Missing	14 (5.39)

**Doing the test was unpleasant.**	***n* (%)**
Disagree/Strongly disagree	154 (59.23)
Agree/Strongly agree	90 (34.62)
Missing	16 (6.15)

**Were you advised of result by GP practice?**	***n* (%)**
No/Don’t know	64 (24.62)
Yes	186 (71.54)
Missing	10 (3.84)

**Did you receive a ‘Positive’ test result?**	***n* (%)**
No	145 (55.77)
Yes	86 (33.08)
Missing	29 (11.15)

*SD = standard deviation.*

Patient satisfaction was generally high. Satisfaction with FIT was highest (87.69% [ *n* = 229] agreed or strongly agreed that their overall impression of the test was satisfactory), followed by the way they received results (75.61% [ *n* = 197] indicated they were satisfied with the way in which they received their results), and the GP consultation (73.85% [ *n* = 192] indicated they were satisfied or very satisfied with their GP consultation) (see Tables 3, 4, and 5, respectively).

**Table 3.** Demographic and psychological variation in satisfaction with GP consultation^a^

**Characteristic**	**Satisfaction,** ***n* (%)**	**OR (95% CIs)**	**aOR (95% CIs)**
All participants (*n* = 260)	**192 (73.85)**		

**Age**			
Age in years (continuous)		1.01 (0.98 to 1.04)	1.00 (0.96 to 1.04)

**Gender**			
Male	80 (72.86)	1.00	1.00
Female	112 (75.57)	1.15 (0.66 to 2.03)	0.84 (0.41 to 1.72)

**Ethnicity**			
White British	184 (74.86)	1.00	1.00
Any other ethnicity	8 (65.57)	0.64 (0.19 to 2.22)	0.46 (0.09 to 2.17)

**Education**			
O Level or GCSE (Grade D G) or lower qualification	92 (79.79)	1.00	1.00
O Level or GCSE (Grade A C) or higher qualification	99 (70.04)	0.60 (0.29 to 1.22)	0.45 (0.17 to 1.22)

**Area-level deprivation**			
IMD tertile 1	55 (84.62)	1.00	1.00
2	57 (80.00)	0.49 (0.19 to 1.25)	0.56 (0.20 to 1.56)
3	65 (58.32)	**0.40 (0.17 to 0.94)**	**0.25 (0.09 to 0.67)^b^**

**Migrant status**			
Born in the UK	179 (74.13)	1.00	1.00
Emigrated to the UK	13 (78.57)	1.31 (0.33 to 5.14)	1.01 (0.24 to 4.26)

**Car ownership**			
No car or van	29 (76.60)	1.00	1.00
One or more car and or van	163 (74.05)	0.87 (0.37 to 2.03)	0.59 (0.18 to 1.94)

**Did your GP explain the purpose of the test to you?**			
No/Don’t know	18 (45.77)	1.00	1.00
Yes	174 (79.71)	**4.66 (2.20 to 9.85)^b^**	**3.55 (1.40 to 9.00)^c^**

**Did the GP provide instructions on how to complete the test?**			
No/Don’t know	69 (67.79)	1.00	1.00
Yes	123 (78.70)	1.76 (0.99 to 3.13)	1.76 (0.81 to 3.80)

**Did the GP tell you how long the test would take to arrive?**			
No/Don’t know	67 (65.81)	1.00	1.00
Yes	125 (79.97)	**2.08 (1.16 to 3.70)^b^**	1.55 (0.71 to 3.38)

a *The percentages in the satisfaction column refer to the percentage of people in each category who were satisfied with the consultation. The denominator in the regression models is 260 as multiple imputation was used to calculate missing data.*

b P*<0.05.*

c P*<0.01. aOR = adjusted odds ratio. IMD = Indices of Multiple Deprivation. CI = confidence interval. OR = odds ratio.*

**Table 4.** Demographic and psychological variation in satisfaction with doing the FIT test

**Characteristic**	**Satisfaction (%)**	**OR (95% CIs)**		**aOR (95% CIs)**
All participants (*n* = 260)	**229 (87.69)**			

**Age**				
Age in years (continuous)		0.99 (0.95 to 1.03)		1.00 (0.95 to 1.05)

**Gender**				
Male	101 (93.52)	1.00		1.00
Female	128 (86.49)	0.57 (0.25 to 1.31)		2.18 (0.79 to 6.09)

**Ethnicity**				
White British	219 (90.12)	1.00		1.00
Any other ethnicity	10 (83.33)	0.63 (0.13 to 3.03)		0.85 (0.08 to 9.65)

**Education**				
O Level or GCSE (Grade D G) or lower qualification	100 (86.36)	1.00		1.00
O Level or GCSE (Grade A C) or higher qualification	129 (90.72)	1.45 (0.67 to 3.57)		1.66 (0.49 to 5.69)

**Area-level deprivation**				
IMD tertile 1	60 (92.31)	1.00	1.00	
2	64 (90.14)	0.66 (0.23 to 0.76)		0.53 (0.14 to 2.08)
3	81 (85.26)	0.18 (0.17 to 0.48)		0.32 (0.09 to 1.15)

**Migrant status**				
Born in the UK	213 (91.02)	1.00		1.00
Emigrated to the UK	16 (88.88)			

**Car ownership**				
No car or van	31 (81.38)	1.00		1.00
One or more car and or van	198 (90.02)	2.06 (0.81 to 5.26)		0.97 (0.25 to 3.72)

**Instruction scale**				
Instruction scale (continuous)		1.17 (0.97 to 1.42)		1.29 (0.95 to 1.75)

**Collection scale**				
Collection scale (continuous)		1.06 (0.92 to 1.23)		1.12 (0.88 to 1.41)

**Doing the test made me feel anxious**				
Disagree/Strongly disagree	131 (83.97)	1.00		1.00
Agree/Strongly agree	98 (96.08)	**4.67 (1.60 to 14.01)^a^**		**5.99 (1.28 to 28.09)^a^**

**The thought of an abnormal result**				
**from the test scared me**				
Disagree/Strongly disagree	73 (82.92)	1.00		1.00
Agree/Strongly agree	156 (91.77)	**2.30 (1.05 to 5.02)^a^**		1.66 (0.60 to 4.61)

**Doing the test was unpleasant**				
Disagree/Strongly disagree	139 (84.72)	1.00		1.00
Agree/Strongly agree	90 (95.76)	**4.08 (1.37 to 12.14)^a^**		3.19 (0.88 to 11.51)

a P*<0.05. aOR = adjusted odds ratio. FIT = faecal immunochemic al testing. IMD = Indices of Multiple Deprivation. CI = confidence interval. OR = odds ratio.*

**Table 5.** Demographic and psychological variation in satisfaction with receiving results

**Characteristic**	**Satisfaction (%)**	**OR (95% CI)**	**aOR (95% CI)**
All participants (*n* = 260)	**197 (75.61)**		

**Age**			
Age in years (continuous)		1.02 (0.99 to 1.05)	1.01 (0.99 to 1.06)

**Gender**			
Male	86 (77.96)	1.00	1.00
Female	111 (74.90)	0.84 (0.46 to 1.60)	0.79 (0.34 to 1.85)

**Ethnicity**			
White British	186 (75.67)	1.00	1.00
Any other ethnicity	11 (86.89)	2.25 (0.30 to 16.64)	1.89 (0.11 to 33.67)

**Education**			
O Level or GCSE (Grade D–G) or lower qualification	92 (79.79)	1.00	1.00
O Level or GCSE (Grade A–C) or higher qualification	104 (73.28)	0.69 (0.36 to 1.35)	0.56 (0.21 to 1.51)

**Area-level deprivation**			
IMD tertile 1	56 (85.85)	1.00	1.00
2	53 (75.20)	0.50 (0.19 to 1.25)	**0.21 (0.06 to 0.69)^a^**
3	67 (70.95)	**0.40 (0.17 to 0.94)^a^**	**0.19 (0.06 to 0.62)^a^**

**Migrant status**			
Born in the UK	182 (75.54)	1.00	1.00
Emigrated to the UK	14 (85.71)	2.27 (0.20 to 26.17)	4.50 (0.28 to 72.76)

**Car ownership**			
No car or van	28 (75.00)	1.00	1.00
One or more car and or van	168 (76.41)	1.08 (0.42 to 2.73)	1.12 (0.34 to 3.61)

**Were you advised of result by GP practice?**			
Yes	166 (86.55)	1.00	1.00
No	31 (46.22)	**0.13 (0.07 to 0.26)^b^**	**0.09 (0.03 to 1.09)^b^**

**How soon after returning the test did you receive a result?**			
Number of days		0.93 (0.87 to 1.00)	0.98 (0.88 to 1.09)

**Did you receive a ‘Positive’ test result?**			
Yes	67 (73.10)	1.00	1.00
No	123 (80.26)	1.61 (0.84 to 3.09)	1.78 (0.77 to 4.13)

a P*<0.05.*

b P*<0.01. aOR = adjusted odds ratio. CI = confidence interval. IMD = Indices of Multiple Deprivation. OR = odds ratio.*

### Patient satisfaction with the GP consultation

In the univariate analysis, significantly fewer people were satisfied with the GP consultation in the most deprived tertile of areas compared with the least deprived tertile of areas (68.84% versus 84.62%, odds ratio [OR] 0.40, 95% confidence interval [CI] = 0.17 to 0.94, *P*<0.05; there was no statistically significant difference in satisfaction between people living in the most deprived tertile of areas and those living in the median tertile of areas [58.32% versus 80.00%, *P*>0.05]). The GP explaining the purpose of the test (79.71% and 45.77%, OR 4.66, 95% CI = 2.20 to 9.85, *P*<0.05) and how long the FIT kit would take to arrive (79.97% and 66.81%, OR 2.08, 95% CI = 1.16 to 3.70, *P*<0.05) were associated with increased satisfaction with the GP consultation.

Results were similar for the multivariate analysis, with significantly fewer adults in the most deprived tertile of areas being satisfied with the GP consultation compared with the least deprived tertile of areas (68.84% versus 84.62%; adjusted odds ratio [aOR] 0.25, 95% CI = 0.09 to 0.67, *P*<0.05; again there was no statistically significant difference in satisfaction between people living in the most deprived tertile of areas and those living in the median tertile of areas [58.32% versus 80.00%, *P*>0.05]), and whether the GP explained the purpose of the test remained associated with increased satisfaction with the GP consultation (46% versus 80%, aOR 3.55, 95% CI = 1.40 to 9.00, *P*<0.05). The GP explaining how long the test would take to arrive, however, was no longer a statistically significant predictor (*P*>0.05).

### Patient satisfaction with doing the test

In univariate analysis, finding the thought of an abnormal test result *‘*scary’ (92% versus 83%, OR 2.30, 95% CI = 1.05 to 5.02, *P*<0.05), feeling anxious when doing the test (96% versus 84%, OR 4.67, 95% CI = 1.6 to 14.01, *P*<0.05), and perceiving doing the test as ‘unpleasant’ (96% versus 85%, OR 4.08, 95% CI = 1.37 to 12.14, *P*<0.05) were significantly associated with increased satisfaction with doing the test.

However, in the multivariate analysis, only greater anxiety when doing the test remained statistically significantly associated with increased satisfaction with completing the test (aOR 5.99, 95% CI = 1.28 to 28.09, *P*<0.05).

### Patient satisfaction with receiving the results

In the univariate analysis, significantly fewer people were satisfied with how they received their results in the most deprived tertile of areas, compared with the least deprived tertile of areas (70.95% versus 85.85%, OR 0.40, 95% CI = 0.17 to 0.94, *P*<0.05; there was no statistically significant difference in satisfaction between people living in the most deprived tertile of areas and those living in the median tertile of areas [70.95% versus 75.20%, *P*>0.05]). Similarly, not being advised of the result directly by the GP, or having to actively seek the result themselves, were associated with decreased satisfaction with receiving the results (46% versus 87% respectively, OR 0.13, 95% CI = 0.07 to 0.26, *P*<0.05).

Results were similar in the multivariate analysis, with significantly fewer people in the most deprived tertile of areas being satisfied with receiving the results compared with the least deprived tertile of areas (70.95% versus 85.85%, aOR 0.19, 95% CI = 0.06 to 0.62, *P*<0.05; significantly fewer people in the most deprived tertile of areas were also satisfied when compared with those in the median deprived tertile of areas [70.95% versus 75.2%, aOR 0.21, 95% CI = 0.06 to 0.69]), and not being advised of the result directly being statistically significantly associated with decreased satisfaction with receiving the results (46% versus 87%, aOR 0.19, 95% CI = 0.06 to 0.62 and aOR 0.09, 95% CI = 0.03 to 0.27, *P*<0.05 respectively).

### Study 2: Semi-structured interviews

Of the 260 adults who returned a survey, 60 (23.1%) consented to participate in a follow-up interview. Of these, 20 (7.7%) were ultimately selected (at random) and contacted for interview.

The mean age of participants participating in interviews was 63.5 years (range 37–78) and the mean IMD tertile of the population was 2.13 (range 1–3).

The majority of participants were female (*n* = 11, 55.0%), identified as white British (*n* = 17, 85.0%), had obtained a GCSE, O Level (Grade A–C), or higher qualification (*n* = 16, 80.0%), were born in the UK (*n* = 16, 80.0%), and owned one or more cars and/or vans (*n* = 16, 80.0%) (Table 6).

**Table 6.** Sample characteristics of interview participants

**Age**	**Mean (Range)**
Age in years (continuous)	63.47 (37 78)

**Gender**	***n* (%)**
Male	6 (30.00)
Female	11 (55.00)
Missing	3 (15.00)

**Ethnicity**	***n* (%)**
White British	17 (85.00)
Any other ethnicity	0
Missing	3 (15.00)

**Education**	***n* (%)**
O Level or GCSE (Grade D G) or lower qualification	1 (5.00)
O Level or GCSE (Grade A C) or higher qualification	16 (80.00)
Missing	3 (15.00)

**Area-level deprivation**	**Mean (Range)**
IMD tertile (continuous)	2.13 (1 3)

**Migrant status**	***n* (%)**
Born in the UK	17 (85.00)
Emigrated to the UK	0
Missing	3 (15.00)

**Car ownership**	***n* (%)**
No car or van	1 (5.00)
One or more car and or van	16 (80.00)
Missing	3 (15.00)

*IMD = Indices of Multiple Deprivation.*

#### Satisfaction with GP consultation.

The interviews explored issues related to satisfaction with the GP consultation and revealed insights otherwise unobtainable by quantitative measures (see Supplementary Box S1 for themes and related quotes). One of the issues explored in more detail was ‘whether the GP explained the purpose of the test’. This was something that was found to be a significant predictor of satisfaction in Study 1, but the reasons as to why it was important were not measured or explored. The present analysis revealed that ‘not knowing the purpose of the test’ caused patients to feel anxious and confused, as they did not know what the test was for or what conditions the GP might be concerned about. In addition, not explaining the purpose of the test resulted in some patients feeling confused about whether they needed to do the test, and this was particularly true if they had been given more than one stool test to complete or had completed stool tests in the past (for example, for screening purposes).

Several other issues concerning the GP consultation manifested naturally during the interviews but could not be linked to the results of Study 1. Pertinent among these was the (perceived) impersonal nature of telephone consultations, as well as the technological issues experienced with them. Where face-to-face appointments were possible, patients often stated they felt as though *‘GPs were rushing’*.

#### Patient satisfaction with doing the test

When it came to completing the test, anxiety was found to be a significant predictor of satisfaction in the multivariate analysis and was therefore explored further in the interviews. Patients who reported anxiety completing the test discussed both family history of cancer and heightened concerns about bowel cancer, and subsequently described feeling reassured by the ‘peace of mind’ the test provided. Patients also reported feeling anxious about how much sample was needed, and whether enough had been provided. Related to this were issues with reinserting the applicator stick into the sample tube, which patients explained was difficult owing to the narrow opening of the test tube, and added to the difficulty and unpleasantness of performing the test.

#### Patient satisfaction with receiving results

Finally, three key themes emerged in relation to satisfaction with receiving the results. First and foremost was the importance of directly receiving a test result. Several individuals explained that the reason for this was that they never received their result, and ‘felt let down’ by this. This sense of disappointment was expressed even where patients were informed that they would not hear from the GP if the result was normal, as ‘there’s always incompetence’. Patients wanted to receive their results from their GP, ideally face-to-face, and not from ‘the receptionist’ (where a result was not received, patients would have been content with a text message). Finally, where the test result was normal, patients felt reassured that it was not bowel cancer, but were dissatisfied that there was no diagnosis and no further follow-up regarding their symptoms.

## DISCUSSION

### Summary

This study explored patient satisfaction with three key aspects of the symptomatic FIT pathway: the GP consultation, completing the FIT kit, and receiving the test results. Overall, satisfaction was highest for completing the FIT kit, followed by the way the FIT result was explained, and finally the GP consultation. Several factors were found to be significantly associated with satisfaction for each.

With regards to the GP consultation, area-level deprivation and whether the GP explained the test purpose were found to be predictors of satisfaction in the multivariate analysis, with increased area-level deprivation associated with decreased satisfaction and clear explanation of the test purpose associated with increased satisfaction. For satisfaction with doing the test, anxiety completing the kit was statistically significantly associated with increased satisfaction after adjusting for covariates. Finally, in terms of satisfaction with receiving the results, both increased area-level deprivation and the patient not being directly advised of their test result were independently associated with decreased satisfaction at the *P*<0.05 threshold.

Follow-up interviews provide insights into these findings. For the first issue ‘how does the GP not explaining the purpose of the FIT test lead to dissatisfaction’, two underlying reasons were identified. The first was that it resulted in anxiety, with patients expressing that more information, particularly about what the test was ‘looking for’, would have provided greater reassurance. The second was that it led to confusion, particularly where patients had previously participated in bowel cancer screening and felt that the need for a second test was concerning. In relation to understanding ‘increased anxiety when completing the test’, one possible explanation as to why participants who reported higher anxiety also reported higher satisfaction did emerge. Specifically, it was found that participants who reported higher anxiety in the survey often explained that they were anxious about doing the test, as they had a family history of bowel cancer, and that this anxiety was later alleviated by the news that their test result was negative.

Finally, with respect to the third issue ‘why receiving the results through other means leads to dissatisfaction with receiving the results’, three concerns were identified. First, patients reported that they did not receive a result, and were concerned this may have been due to ‘incompetence’, as opposed to the result not being reported as it was ‘normal’. Second, not hearing from the GP meant there was a lack of diagnosis and follow-up, despite symptoms persisting. Third, where results were provided by a receptionist, there was similarly no diagnosis or follow-up, and a sentiment that receptionists were not qualified to provide a detailed explanation of the implications of the results.

### Strengths and limitations

This study has several strengths. First, the survey was administered 2–3 weeks after the participant’s FIT kit, minimising recall bias, which is often associated with longer intervals between events and data collection.^20^ Second, an explanatory mixed-methods approach was used, which allowed the authors to explore the issues identified in the questionnaire in detail.^21^ Finally, following each stage of qualitative data analysis, two reviewers discussed the thematic findings and resolved disagreements through discussion, maintaining theoretical validity.^22^

This study had several limitations. First, it used self-sampling to recruit participants, and, as it did not have consent to access data on non-responders to the questionnaire, it could not determine whether the data was subject to ‘self-selection bias’. Second, the relatively low uptake rate of 16.55% for the survey, and 23.08% for subsequent interviews, may affect the representativeness of the results (again, as consent to access data on non-responders was not given, it was not possible to confirm this). Third, the study was conducted with patients who were offered symptomatic FIT during lockdown, when most patients were offered a telephone consultation, affecting the generalisability of the findings. Fourth, the survey was written and distributed in English, meaning only recipients who were able to read and write English were able to complete the survey (the sample was >93% white British, which while representative of St Helens and Knowsley is not representative of England’s population, which is 86% white.^23^ Further research is required to understand the experience and satisfaction with symptomatic FIT in black and minority ethnic communities, who may have different experiences of the health system, as documented previously.^24^ Fifth, the survey did not include a comprehensive range of individual-level measures for socioeconomic deprivation (exclusions include home ownership and household income), and instead used a combination of postcode, car ownership, and educational attainment to determine socioeconomic status.

Finally, while St Helens and Knowsley represent two of the most deprived boroughs in England, the higher reported educational attainment and IMD scores suggest that the least deprived were overrepresented within the study sample. This may reflect self-sampling bias, or it may reflect recent findings which suggest that individuals from more socioeconomically deprived backgrounds were less likely to seek help for possible CRC symptoms (and therefore to complete this survey) during the COVID-19 pandemic.^25^

### Comparison with existing literature

The findings of this study are consistent with those exploring patient satisfaction with FIT in other contexts. For example, in a study assessing the use of FIT as a yearly surveillance mechanism for patients at high risk of CRC, individuals reported high satisfaction with completing the FIT kit.^26^ Similarly, a recent study using survey data to explore usability and acceptability of FIT for symptomatic adults also found that most patients reported high test acceptability.^27^ These findings build upon the research by assessing patient satisfaction with the entire primary care FIT pathway, from GP consultation to completing the kit and receiving the results. Further, the explanatory mixed-methods approach was able to qualitatively explore issues relating to satisfaction with each aspect of the pathway, resulting in clear implications for improvements (not just with the test, but also the consultation and delivery of results). The finding that consultation satisfaction is lower in more socioeconomically deprived areas is also consistent with previous literature, which suggests that satisfaction with consultations is lower in these regions owing to a wide range of factors, including increased demand and shorter consultation length.^28^^–^30

### Implications for research and practice

The results of this study highlight that satisfaction with the FIT test is very high, while satisfaction with the GP consultation and the way the FIT result is explained are comparatively low. As such, efforts to improve patient satisfaction with the symptomatic FIT pathway should focus more on the initial GP consultation and the way the test and test result are explained, and less on modifications to the test kit and accompanying instructions.

With regards to improving patient satisfaction with the way the FIT result is explained, the most evident step would be to ensure that patients always receive their result, irrespective of whether it is normal or abnormal. Based on patient feedback, there is a preference for the result to be communicated by the GP (and not the receptionist), given that it relates to cancer. One possible solution, suggested by patients, would be for GPs to text them their results (when results are normal).

In terms of the GP consultation, decreased satisfaction in more deprived areas may well be explained by reduced consultation times seen in these areas^30^ caused by increased patient practice lists, demand for services (that is, poorer health of populations in these areas),^29^ and subsequent GP workload. These shorter consultations may result in less opportunity for questions and clarification (explaining the purpose of the test was shown to be important in this study), and thereby increased confusion and anxiety about the test (as observed in the interviews). Improving the clarity of the instructions, and providing further details about the test, how to catch the bowel motion, etc. could help address some of these issues. This relationship between deprivation and satisfaction is important and warrants further investigation; future research should focus on understanding dissatisfaction in more deprived regions.

Finally, there was no access to data on completion rates within this study. However, previous research investigating the diagnostic accuracy of FIT in patients referred by their GP for suspected CRC found that 23.50% either did not return their kit (22.79%) or returned it in an unusable state (0.71%). This has implications for British Society of Gastroenterology safety-netting guidelines, which currently differ based on whether a patient is deemed to be low or high risk by their GP, and whether a TWW referral has been made. Measures include informing patients and GPs of failure to complete, the NHS trust contacting patients directly to encourage completion, and a clinical review and triage based on referral information to determine priority level followed by telephone contact by an appropriately trained clinician. Further research is needed to understand why some patients do not return their kit, and thereby enable the development of interventions to reduce non-completion rates.

## Data

All data files are available via Open Science Framework (https://osf.io/tv23f/).
